# Evaluation of Zn^2+^- and Cu^2+^-Binding Affinities of Native Cu,Zn-SOD1 and Its G93A Mutant by LC-ICP MS

**DOI:** 10.3390/molecules27103160

**Published:** 2022-05-15

**Authors:** Julia Smirnova, Julia Gavrilova, Andra Noormägi, Karin Valmsen, Hegne Pupart, Jinghui Luo, Vello Tõugu, Peep Palumaa

**Affiliations:** 1 Department of Chemistry and Biotechnology, Tallinn University of Technology, Akadeemia tee 15, 12618 Tallinn, Estonia; julia.smirnova@taltech.ee (J.S.); julia.gavrilova1@gmail.com (J.G.); andra.noormagi@gmail.com (A.N.); karin.valmsen@gmail.com (K.V.); hegne.pupart@gmail.com (H.P.); vello.tougu@taltech.ee (V.T.); 2 Paul Scherrer Institute, Forschungsstrasse 111, 5232 Villigen, Switzerland; jinghui.luo@psi.ch

**Keywords:** Cu,Zn-SOD1, ALS, metal-binding affinity, LC-ICP MS

## Abstract

The tight binding of Cu and Zn ions to superoxide dismutase 1 (SOD1) maintains the protein stability, associated with amyotrophic lateral sclerosis (ALS). Yet, the quantitative studies remain to be explored for the metal-binding affinity of wild-type SOD1 and its mutants. We have investigated the demetallation of Cu,Zn-SOD1 and its ALS-related G93A mutant in the presence of different standard metal ion chelators at varying temperatures by using an LC-ICP MS-based approach and fast size-exclusion chromatography. Our results showed that from the slow first-order kinetics both metal ions Zn^2+^ and Cu^2+^ were released simultaneously from the protein at elevated temperatures. The rate of the release depends on the concentration of chelating ligands but is almost independent of their metal-binding affinities. Similar studies with the G93A mutant of Cu,Zn-SOD1 revealed slightly faster metal-release. The demetallation of Cu,Zn-SOD1 comes always to completion, which hindered the calculation of the *K_D_* values. From the Arrhenius plots of the demetallation in the absence of chelators Δ*H*^‡^ = 173 kJ/mol for wt and 191 kJ/mol for G93A mutant Cu,Zn-SOD1 was estimated. Obtained high ΔH values are indicative of the occurrence of protein conformational changes before demetallation and we concluded that Cu,Zn-SOD1 complex is in native conditions kinetically inert. The fibrillization of both forms of SOD1 was similar.

## 1. Introduction

Cu,Zn-superoxide dismutase 1 (SOD1) is a cytoplasmic antioxidant enzyme, important for defense against potentially toxic superoxide radicals [1], produced as natural by-products in aerobic respiration [2]. SOD1 is expressed as a 16 kDa polypeptide chain, whose maturation into an enzymatically active protein involves metalation by Zn^2+^- and Cu^+^ ions, the formation of a conserved intra-subunit disulfide bond between Cys-57 and Cys-146, and subsequent dimerization [1]. Metalation of SOD1 with a copper ion is assisted by a copper chaperone for SOD-CCS, which also participates in the formation of a disulphide bond, uncommon for cytosolic proteins [3,4]. The redox-active copper ion is responsible for the catalytic activity in the conversion of superoxide radicals to hydrogen peroxide and dioxygen [5,6]. It is suggested that Zn^2+^ ion contributes to the formation and persistence of the native structure as its removal leads to immediate inactivation of SOD1 [7]. The major structural motif of SOD1 is β-barrel, whereas the metal-binding site is composed of loop regions located at the bottom of the active site channel [8].

SOD1 is characterized by extreme thermal stability and resistance to chemical denaturation. The melting temperature of natively folded Cu,Zn-SOD1, as measured by differential scanning calorimetry (DSC), is between 92 °C and 101 °C [5,9,10]. The remarkable stability of SOD1 is supported by several factors. The main contribution comes from the binding of metal ions, as apo-protein has substantially lower stability [9,11,12]. The redox state of copper is also important: the reduction of Cu^2+^ ions to Cu^+^ with dithionite increases the thermal stability of bovine Cu,Zn-SOD1 protein by four degrees [13]. The protein is further stabilized by a conserved intra-subunit disulfide bond anchoring the loop that forms part of the dimer interface to the β-barrel [5]. The dimerization also greatly increases the stability of Cu,Zn-SOD1 by reducing the solvent-accessible surface area [14].

SOD1 is involved in the pathogenesis of amyotrophic lateral sclerosis (ALS), as in the case of 20–25% of the familial ALS (fALS) patients the SOD1 protein is mutated [5]. According to The Human Gene Mutation Database, 209 mutations of SOD1 were identified, both inside and outside of the active site. Accordingly, based on SOD activities and metal-binding properties, two groups of fALS SOD1 mutants were distinguished: the metal-binding region (MBR) mutants and the wild-type-like (WTL) mutants [5]. In the case of the MBR mutants of SOD1, abolished or distorted binding of metal ions leads to the loss of native catalytic activity. Moreover, misbound copper ions can catalytically generate reactive oxygen species (ROS) through Fenton or Haber–Weiss chemistry, thus gaining a toxic function. Besides catalytic incompetence, it is known that the ALS mutants of SOD1 can distort the structure of SOD1 into conformations prone to aggregation and fibrillization, which is also observed in the case of ALS [1,15]. Those SOD1 aggregates can also bind copper ions and gain toxic function, as described above. The WTL mutants of SOD1 are remarkably similar to wild-type SOD1 in most of their physico-chemical properties, nonetheless, often these variants were shown to aggregate more readily [16].

**Figure 1:**
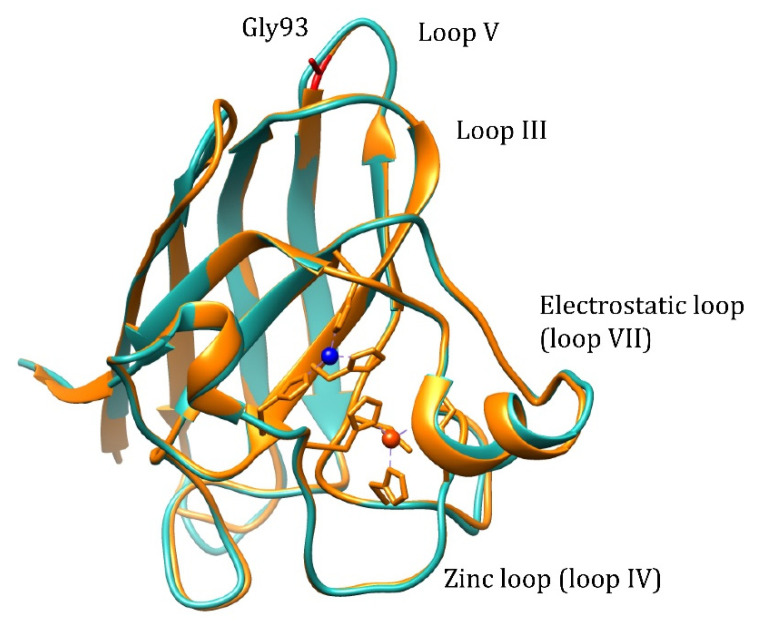
Structure alignment of the human wt Cu,Zn-SOD1 (light-blue, PDB: 2C9V) and G93A mutant (yellow, PDB: 2WKO). Cu^2+^ ion: blue, Zn^2+^ ion: orange.

Pathogenic WTL mutation G93A is located in loop V where it is distant from the metal binding region, disulfide, and dimer interface regions, but is conserved in 90% of SOD1 sequences (Figure 1). Mutation retains metal binding and catalytic activity, but rotates the peptide bond carbonyls of residues 92 and 93 away from the core and shifts the overall position of loop V away from loop III [17]. Based on NMR data, it is demonstrated that the remote metal-binding region is also selectively destabilized by G93A mutation [18], which should reflect in the changes of the metal-binding properties of the protein. The transgenic mice expressing human G93A Cu,Zn-SOD1 show progressive aggregation of the G93A Cu,Zn-SOD1 and ubiquitin in the spinal cord motor neurons [19,20], which points towards an increased propensity to aggregation for the G93A mutant. Thus, it is possible to explain the pathogenicity of the G93A mutation by changed metal-binding properties or increased propensity to aggregation.

Discrimination between these two possibilities could be realized by in vitro studies of the metal-binding properties and fibrillization of wt Cu,Zn-SOD1 and its G93A mutant. Despite the obvious importance of SOD1 interaction with metal ions, there are still no reliable quantitative data about the metal-binding affinity of wt Cu,Zn-SOD1. It is known that the metal-binding affinity of SOD-1 is very high in the native conditions, which is common for the binding sites buried in the protein interior. There is also evidence that the metal release from wt Cu,Zn-SOD1 might be connected with the unfolding of the protein [21]. So far there are attempts to estimate the K_D_ values for the metal-SOD1 complexes only under mildly denaturing conditions. In these studies, the competition of the partially denatured protein with the metal chelators of known metal-binding affinity was studied [22]. These results cannot be used for the estimation of the metal-binding affinity of SOD1 in native conditions, which is needed for the comparison with the metal-binding properties of wt and mutated SOD1 forms.

To get quantitative information about the metal-binding affinities of wt and G93A mutant SOD1, we have investigated the demetallation of corresponding proteins at elevated temperatures in the presence of different standard metal ion chelators by using an LC-ICP MS-based approach, elaborated in our previous study [23]. Size exclusion chromatography was used to separate high molecular weight (HMW) SOD1 fraction from low molecular weight (LMW) metal complexes, which gives an adequate reflection of metal ion release. Such a direct approach showed that both metal ions Zn^2+^ and Cu^2+^ are released simultaneously at elevated temperatures from the protein, according to slow first-order kinetics. The rate of the release depends on the concentration of chelating ligands, but is almost independent of their metal-binding affinities. The obtained results demonstrate that the metal ion release from the wt Cu,Zn-SOD1 is dependent on the rate-limiting opening of the native protein conformation, where chelating ligands can form ligand-exchange complexes for the release of metal ions without preference. As the demetallation of Cu,Zn-SOD1 always comes to completion, it is impossible to calculate the metal-binding affinities of native SOD1 for Zn^2+^ and Cu^2+^ ions and we have to conclude that the Cu,Zn-SOD1 complex is kinetically inert in native conditions. By using concentration dependences and Arrhenius plots, we estimated that the half-life for the dissociation of metal ions from native Cu,Zn-SOD1 at 40 °C is approximately 3.5 months. Similar studies with the G93A mutant of Cu,Zn-SOD1 revealed slower metal-release and higher thermodynamic stability of mutant SOD1 at physiological temperatures, whereas the fibrillization of both forms of SOD1 was similar.

## 2. Results

### 2.1. Preparation of Cu,Zn-SOD1

After expression and purification, the human SOD1 protein with MW of 15,805.2 Da determined by MALDI TOF MS (theoretical molecular weight 15,804.5 Da) and the G93A mutant with MW of 15,817.6 Da were obtained. ICP-MS analysis demonstrated that both SOD-1 forms were fully metalated with Cu and Zn. From 20 g of bacterial mass, 13.8 mg of pure wt Cu,Zn-SOD1 protein was obtained, and 80 mg of Cu,ZnSOD-1(G93A) was obtained from 60 g of bacterial mass.

### 2.2. Demetallation of Cu, ZnSOD1 at Elevated Temperatures in the Presence of EDTA

**Figure 2:**
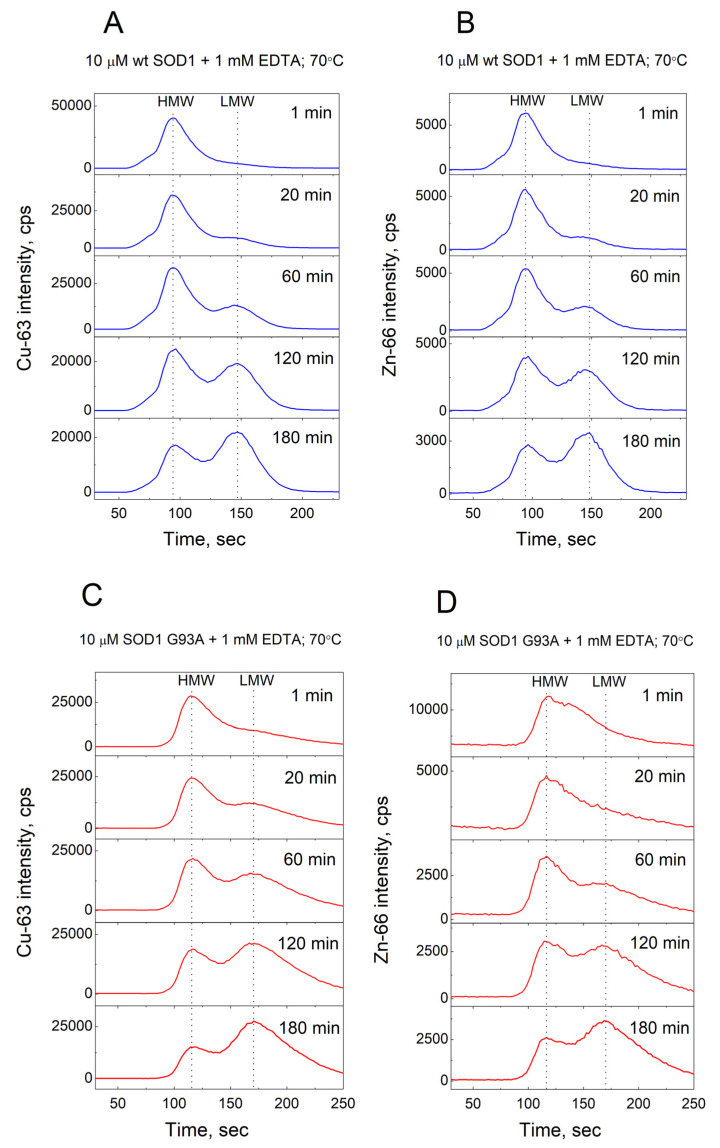
Demetallation of wt and G93A mutant Cu,Zn-SOD1 monitored by LC-ICP-MS. Conditions: 10 µM wt Cu,Zn-SOD1 (**A**,**B**) and 10 µM of its G93A mutant (**C**,**D**) in the presence of 1 mM EDTA at 70 °C in 50 mM HEPES/50 mM NaCl, pH 7.3.

Demetallation of 10 µM wt Cu,Zn-SOD1 was monitored at 60 °C, 70 °C, and 80 °C in the presence of 0, 1, 10, and 50 mM EDTA. The monitored isotopes were Cu-63 and Zn-66. The high and low-molecular-weight metal pools containing protein-metal and EDTA-metal complexes, respectively, were separated by a Sephadex G25 Superfine 1 mL column and the metal content was continuously monitored by ICP MS. Examples of the demetallation of Cu,Zn-SOD1 and its G93A mutant in the presence of 1 mM EDTA at 70 °C are presented in Figure 2.

**Figure 3:**
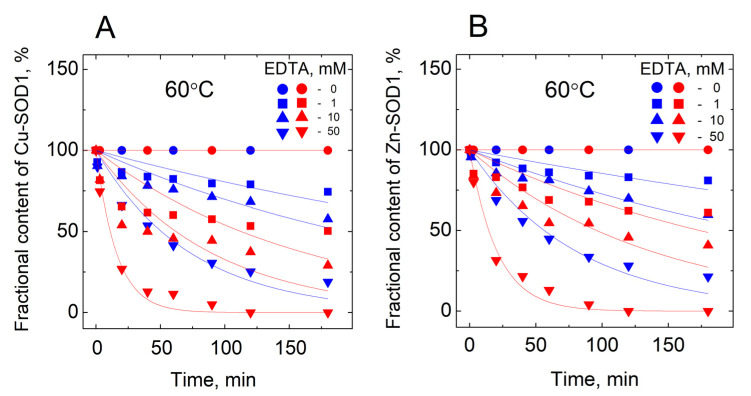
Demetallation of wt Cu,Zn-SOD1 and its G93A mutant in the presence of EDTA monitored by LC-ICP-MS. (**A**) fraction of Cu bound to-SOD1; (**B**) fraction of Zn bound to SOD1. Conditions: 10 µM wt Cu,Zn-SOD1 (blue symbols) and 10 µM of its G93A mutant (red symbols) in 50 mM HEPES/50 mM NaCl, pH 7.3 at 60 °C.

The separation of the HMW and LMW peaks allows integration using Origin software. Kinetic curves reflecting a decrease of the fractional content of metalated protein in time are presented in Figure 3.

**Figure 4:**
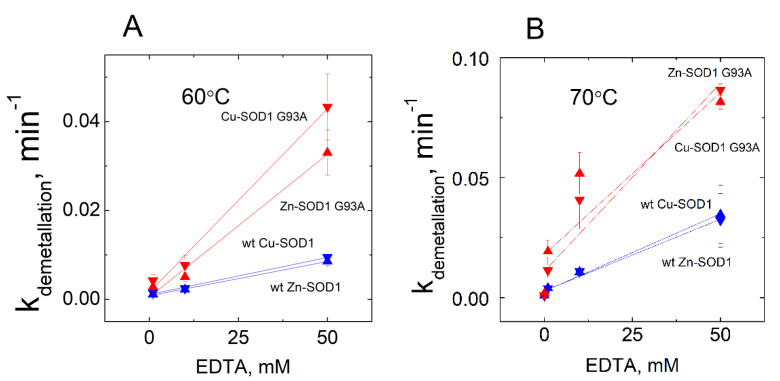
Dependence of the rate constant of demetallation of wt Cu,Zn-SOD1 and its G93A mutant on the EDTA concentration. Demetallation of wt Cu,Zn-SOD1 (blue symbols) and Cu,Zn-SOD1 G93A mutant (red symbols) at 60 °C (**A**) and 70 °C (**B**). Conditions: 10 µM wt Cu,Zn-SOD1 and 10 µM of its G93A mutant in 50 mM HEPES/50 mM NaCl, pH 7.3.

The release of both the copper and zinc occurred concomitantly in all of the conditions shown in the Figure. From the kinetic curves, first-order rate constants were calculated, and their dependencies from the concentration of EDTA are presented in Figure 4.

In the presence of DTPA, which has in comparison with EDTA more than 100 times higher binding affinity for Cu(II) and Zn(II) ions, only two times faster metal release was observed (data not shown).

**Figure 5:**
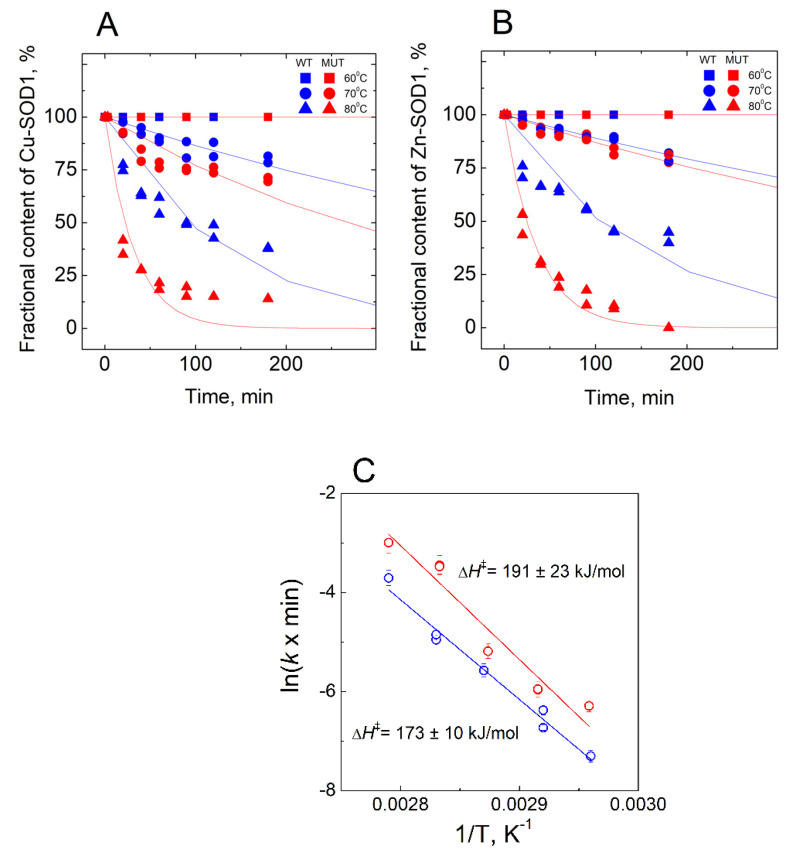
Demetallation of wt Cu,Zn-SOD1 and of its G93A mutant in the absence of EDTA monitored by LC-ICP-MS. (**A**) fraction of Cu bound to SOD1; (**B**) fraction of Zn bound to SOD1; (**C**) Arrhenius plots for the de-coppering of wt and G93A mutant Cu,Zn-SOD1. Conditions: 10 µM wt Cu,Zn-SOD1 (blue symbols) and 10 µM of its G93A mutant (red symbols) in 50 mM HEPES/50 mM NaCl, pH 7.3 at 60 °C, 70 °C and 80 °C. EDTA at 1mM concentration was added to the samples before LC to obtain the LMW peak.

In the absence of EDTA, a release of the metal ions was monitored in temperatures ranging from 60 °C to 85 °C (Figure 5). Experiments at 70 °C and 80 °C were performed in duplicates and the average difference between the parallel measurements was three fractional contents %.

### 2.3. Fibrillization of Cu,ZnSOD-1 and Its G93A Mutant

**Figure 6:**
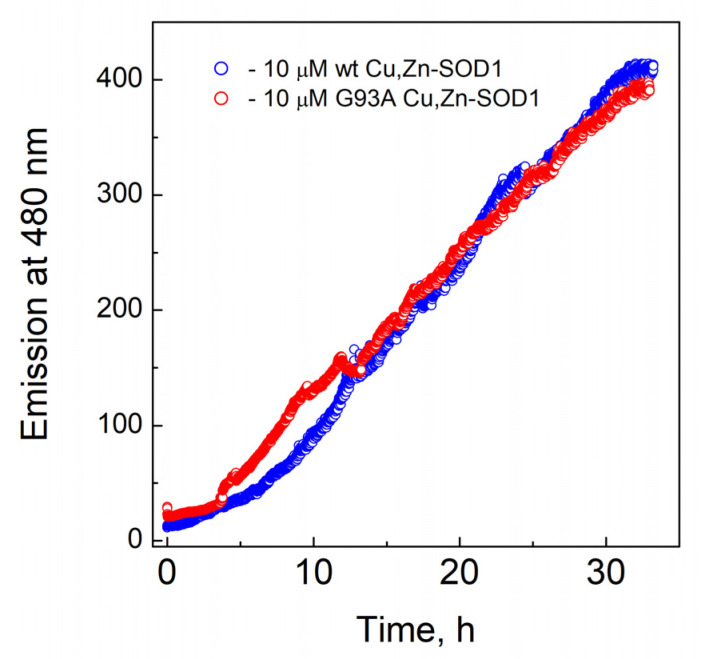
Fibrillization kinetics of Cu,Zn-SOD1. Conditions: 10 µM wt Cu,Zn-SOD1 (blue) and 10 µM of its G93A mutant (red) in 50 mM HEPES/50 mM NaCl, pH 7.3 at 40 °C in the presence of 3.3 µM ThT.

Fibrillization of Cu,Zn-SOD1 and its G93A mutant at 40 °C are presented in Figure 6, which demonstrates that both proteins exposed similar fibrillization kinetics.

## 3. Discussion

The major focus of the study was directed towards the determination of the Cu^2+^ and Zn^2+^ -binding affinities of wt SOD1, and its G93A mutant through competition with high-affinity metal-binding ligands, such as EDTA and DTPA. Demetallation of proteins was monitored by using the LC-ICP MS technique, allowing simultaneous and specific detection of the release for both copper and zinc ions. None of the ligands was able to demetallate these proteins at physiological temperatures, even at high millimolar concentrations, indicating either very high metal-binding affinity or the kinetic inertness of Cu,Zn-SOD1. Demetallation was also studied at elevated temperatures, where Cu^2+^ and Zn^2+^ ions were released from protein simultaneously and the process followed first-order kinetics until full demetallation. The rate of demetallation depended on the ligand concentration, but not on its affinity. Such behavior indicates that the rate-limiting step in the metal release is the opening of the active site to the ligand and the ligand-assisted demetallation. The release of metal ions is cooperative—e.g., the release of Cu^2+^ leads to a release of Zn^2+^ or vice versa–thus, it is not possible to determine the affinity of protein towards one of these ions, or to establish which ion is released first. From the Arrhenius plots of the de-coppering in the absence of chelators Δ*H*^‡^ = 173 ± 10 kJ/mol for wt and 191 ± 23 kJ/mol for G93A Cu,Zn-SOD1 was determined. The obtained high Δ*H*^‡^ value is indicative of the occurrence of protein conformational changes before demetallation and confirms that the rate-limiting step in the metal release is indeed thermal denaturation. Those activation energies fall into the range of the reported values for the proteins, as discussed in [24,25]. By extrapolating Arrhenius dependence to 40 °C, we found that in physiological conditions the metal release from wt Cu,Zn-SOD-1 could occur with a half-life of 3.5 month (k_d_ = 4.37 × 10^−6^ min^−1^), and from Cu,ZnSOD-1 (G93A) with a half-life of 2.8 month (k_d_ = 5.45 × 10^−6^ min^−1^). By using these values and considering that metal binding occurs with a speed below the diffusion limit k_a_ = 10^9^ M^−1^s^−1^, we can estimate the minimal values of the dissociation constants *K_D_* for wt Cu,Zn-SOD1 and G93A mutant Cu,Zn-SOD1 of 7.3 × 10^−17^ and 9.1 × 10^−17^ M, respectively. The difference between the estimated dissociation rate constants between wt and mutant Cu,Zn-SOD1 is very small, which apparently does not influence the functioning of the enzyme. In a separate experiment, we established that fibrillization of the native and mutant Cu,Zn-SOD-1 occurs according to a very similar time curve, thus, there are no differences in the fibrillization propensity of these fully metalated protein forms.

All metal-binding ligands EDTA, DTPA, accelerated at elevated temperatures the metal release from wt and G93A Cu,Zn-SOD1 in a dose-dependent manner, which indicates that ligands should form a ternary complex with Cu,Zn-SOD1 in the activated intermediary state of thermal denaturation. This conclusion is confirmed by the fact that all demetallation curves led to full demetallation. The effect of chelators was slightly stronger in the case of the G93A Cu,Zn-SOD1, which might originate from the lower thermal stability of the mutated protein. Therefore, we can conclude that there exists no equilibrium between SOD-1 and free metal ions and metal removal is a part of an irreversible thermal denaturation process. The obtained results indicate that from metal chelator studies it is impossible to determine directly Cu^2+^ and Zn^2+^ -binding affinities for SOD-1, however, we can confirm that metal chelators facilitate metal release through the formation of the ternary complex, which is faster in the case of the Cu,Zn-SOD1 G93A mutant.

## 4. Materials and Methods

### 4.1. Expression and Purification of Cu,Zn-SOD1

Human wild-type Cu,Zn-SOD1 was produced according to the protocol presented in [26], by overexpression of protein in *Escherichia coli* strain BL21 Star (DE3). The plasmid containing human wt SOD1, and the yeast CCS coding sequences were kindly provided by Dr. Jinghui Luo (Paul Scherrer Institute). G93A mutant SOD1 was constructed using QuikChange Site-Directed Mutagenesis Kit (Agilent) and primers Gly93Ala_frw (GAC ACA TCG GCC ACA GCA TCT TTG TCA GCA GTC) and Gly93Ala_rev (GAC TGC TGA CAA AGA TGC TGT GGC CGA TGT GTC). The entire coding region was sequenced to verify the presence of the desired mutation. All the reagents were obtained from Sigma-Aldrich, with some exceptions: tryptone, yeast extract powder, and bacteriological agar were from Lab M. The plasmid minipreparation kit GeneJET, the pre-stained protein ladder PageRuler™ and the protein staining solution PageBlue™ were from Thermo Scientific™. Protease inhibitor cocktail tablets cOmplete™ were from Roche.

Both wt-Cu,Zn-SOD1 and its G93A mutant were induced at OD600 = 0.5–0.6 with 0.5 mM IPTG in the presence of 3 mM CuCl_2_ (Sigma-Aldrich) and 30 µM zinc acetate (Scharlau). After induction, the culture was incubated for 18 h in a shaking incubator at 23 °C and 180 rpm (New Brunswick Scientific C25KC) for protein expression.

Purification of the expressed wt Cu,Zn-SOD1 proceeded, according to the protocol developed by [26], with minor modifications. The cell lysate was heated at 65 °C for 30 min, centrifuged at 24,500× *g* and 4 °C for 35 min (Beckman Coulter Avanti centrifuge, rotor JA-20) and the precipitate was discarded. This was followed by fractional precipitation with ammonium sulfate with salt concentrations of 50%, 60%, and 90% of the saturation After each step the solution was gently rotated for 2 h at 4 °C following incubation and centrifugation at 24,500× *g* and 4 °C for 15 min to separate the supernatant. After the final step, the solution was centrifuged for 30 min under the same conditions. Purification was carried out by size-exclusion chromatography (SEC) followed with ion exchange chromatography (IEX) on the ÄKTA explorer (Amersham Biosciences) chromatography system. For SEC, the HiLoad^TM^ Superdex^TM^ 75 26/60 column (GE Healthcare) was used with buffer 50 mM TRIS-HCl, pH 7.5. The sample containing dissolved pellets was filtered through a 5 µM microfilter before injection into SEC. The SEC was carried out using the following parameters: flow rate 2 mL/min; detection wavelengths 280 and 680 nm; collection at 1 mL fractions. The MALDI MS analysis was used to find the right fractions to collect. Prior IEX injection fractions containing wt-Cu/Zn-SOD1 were diluted 2.5 times and then concentrated. The IEX column used was 5 mL HiPrep^TM^ DEAE FF (GE Healthcare) equilibrated with buffer 20 mM TRIS-HCl, pH 7.5. The elution was carried out at a flow rate 4 mL/min using two step gradients: 20 CV up to 25% and then 5 CV until 100% of buffer B (250 mM NaCl in 20 mM TRIS-HCl, pH 7.5). MALDI-MS was used for the analysis of the fractions. The wtSOD-1 containing fractions were then pooled and dialyzed against 20 mM ammonium acetate pH 7.4 or desalted by the SEC column before lyophilization on Christ Alpha 1-2 LD plus freeze dryer.

Purification of the G93A mutant of Cu,Zn-SOD1 followed a similar protocol, with several exceptions. The protein containing fraction was not heated as the G93A mutant Cu,Zn- SOD1 is more sensitive to high temperatures than wt Cu,Zn-SOD1. The fractional precipitation with ammonium sulphate was not applied either. Purification was conducted with SEC and the obtained fractions were investigated with MALDI MS. The buffer was exchanged to 20 mM ammonium acetate pH 7.4 and samples were lyophilized.

### 4.2. ICP-MS Analysis of Purified Proteins

Ultrapure Type 2 water with a resistivity of 18.2 MΩ/cm, produced by a Merck Direct–Q UV water purification system (Merck KGaA, Darmstadt, Germany), was used for all applications. The trace metal grade HNO_3_ was from Fisher Scientific, and the multi-element calibration standard and the ICP-MS internal standard mix were from Agilent Technologies.

Inductively coupled plasma mass spectrometry (ICP-MS) on Agilent 7800 series instrument was used to measure the metal content in wt Cu,Zn-SOD1 and G93A mutant samples. Metal concentrations were determined by the external calibration method by using multi-element calibration standard solutions in the range of 0.50–50 ppb in 2% trace metal grade HNO_3_. The protein samples were diluted in 2% HNO_3_ to a final concentration of 0.1 μM and 0.3 μM. The measurements were performed in He mode. For the ICP-MS instrument control Agilent MassHunter 4.4 software version C.01.04 was used under the following conditions: RF power 1550 W, nebulizer gas flow 1.03 L/min, auxiliary gas flow 0.90 L/min, plasma gas flow 15 L/min, nebulizer type: MicroMist, isotopes monitored: Cu-63 and Zn-66. The obtained results were analyzed by the program Origin 9 Pro.

### 4.3. Metals Release Followed with LC-ICP-MS

Stock solutions of wt and G93A Cu,Zn-SOD1 were prepared in ultrapure Type 2 water, whereas stock solutions of ethylenediaminetetraacetic acid (EDTA, 99.995% trace metal basis) and diethylenetriaminepentaacetic acid (DTPA), both from Sigma/Merck (Merck KGaA, Darmstadt, Germany), were neutralized with 0.1 M NaOH to pH 7–8 and further diluted into reaction buffer composed from 50 mM HEPES, 50 mM NaCl, pH 7.3. Mobile phase and reagent solutions were prepared daily before the experiment.

Metal release from both 10 µM wt and G93A Cu,Zn-SOD1 was followed using LC-ICP-MS apparatus from an Agilent Technologies (Santa Clara, CA, USA). An Agilent Infinity HPLC system consisting of 1260 series μ-degasser, 1200 series capillary pump, Micro WPS autosampler and 1200 series MWD VL detector, was coupled with Agilent 7800 series ICP-MS instrument. The samples were prepared in the reaction buffer (50 mM HEPES, 50 mM NaCl, pH 7.3) and incubated under different temperatures from 60 to 85 °C. Before measurement, the vials containing the reaction mixture were placed into the LC system autosampler for about a minute and then returned to the heater. To initiate metal release, the chelating ligand EDTA was used at different concentrations. The reaction was followed for 3 h, and, in some cases, up to 48 h. The demetallation of the SEC column before each experiment was conducted by injecting 5 mM EDTA (injection volume was 10 µL for all experiments). Desalting resin Sephadex G25 Superfine (Amersham/GE Healthcare, Buckinghamshire, UK), was used for the SEC separation of the HMW and LMW pools in 1 mL column at the flow rate 0.4 mL/min. The mobile phase in SEC was 200 mM NH_4_NO_3_ at pH 7.5, prepared daily from TraceMetal Grade nitric acid (Fisher Scientific UK Limited, Leicestershire, UK) and ammonium hydroxide 25% solution (Honeywell Fluka, Seelze, Germany), which is compatible with ICP-MS. For instruments’ control and data acquisition, ICP-MS MassHunter 4.4 software Version C.01.04 from Agilent was used under the following conditions: RF power 1550 W; nebulizer gas flow 1.03 L/min; auxiliary gas flow 0.90 L/min; plasma gas flow 15 L/min; nebulizer type: MicroMist; isotopes monitored: Cu-63 and Zn-66. The data were analyzed and visualized using the program Origin 9 Pro.

Metal release in the absence of the EDTA for both wt and G93A Cu,Zn-SOD1 was followed by incubating 10 µM proteins in 50 mM HEPES, 50 mM NaCl, pH 7.3, at a subset of high temperatures (60 °C, 70 °C, 80 °C). Prior to injection into the LC-ICP-MS system, the fraction of 20 µL was collected and cooled on ice. Before the LC-ICP-MS run, 1 mM EDTA was added to get the LMW peak signal corresponding to the released Cu(II) and Zn(II) ions bound to the EDTA. The demetallation of the SEC column before each experiment was conducted by injecting 10 mM EDTA (injection volume was 10 µL for all experiments). The LC-ICP-MS conditions were as described above, and the results were analyzed by the program Origin 9 Pro.

### 4.4. Fluorescence Spectroscopy

In the fibrillization kinetics’ experiments, a freshly prepared stock solution of Cu,Zn-SOD1 and its G93A mutant were diluted to a final concentration of 10 μM in 50 mM Hepes, pH 7.3, and 50 mM NaCl containing 5 μM ThT. A 500 μL sample was incubated in a 0.5-cm-path-length quartz cell, equilibrated at 40 °C and equipped with a magnetic stirrer. The increase in ThT fluorescence was measured at 480 nm using excitation at 440 nm on a Perkin Elmer (Waltham, MA, USA) LS55 fluorescence spectrophotometer and the “fast” stirring regime was used.

## 5. Conclusions

In conclusion, we confirmed that fully metalated Cu,Zn-SOD1 G93A mutant has slightly faster metal release at elevated temperatures in the absence and presence of metal chelators, which indicates that mutation of distant G93 residue has an effect on the metal-binding properties of the enzyme, as suggested in [18]. At the same time, the fully metalated G93A mutant Cu,Zn-SOD1 exposed fibrillization behavior similar to that of the native Cu,Zn-SOD1. Thus, the fibrillization of G93A SOD-1 protein in transgenic mice expressing human G93A SOD-1 could be caused by some conditions existing in in vivo transgenic mice model. SOD-1 is a constitutively highly expressed protein in vivo constituting about 0.5–1% of the soluble protein expressed in brain and spinal cord. However, in the transgenic mouse model that expresses the G93A SOD-1 mutation, SOD protein is about 13% of total cellular protein [27,28]. It is speculated that at such high protein concentration protein is only partially metalated with zinc. Zinc-depleted SOD-1 is known to be pro-apoptotic [29,30] and exhibits increased fibrillization propensity, which both of which can cause neurodegeneration in the transgenic mouse model. It is important to mention that zinc supplementation protects ALS mutant G93A SOD-1 transgenic mice against the toxicity of ALS-associated SOD [31], which suggests that toxicity is connected with zinc deficiency in G93A SOD-1. Considering that the release of Zn from G94A is slow, being only slightly faster than that of the wt enzyme, the appearance of zinc-depleted SOD-1 arises most likely due to its incomplete metalation, rather than from the zinc depletion from the fully metalated G94A SOD-1.

## Data Availability

The data presented in this study are available on request from the corresponding author.
